# Biofunctionalization of chalcogenide glass fiber to enhance real time and label free detection by mid infrared spectroscopy

**DOI:** 10.1038/s41598-025-06043-4

**Published:** 2025-07-01

**Authors:** Rayan Zaiter, Frédéric Adamietz, Frédéric Désévédavy, Clément Strutynski, Damien Bailleul, Frédéric Smektala, Thierry Buffeteau, Luc Vellutini, Marc Dussauze

**Affiliations:** 1 Institut des Sciences Moléculaires, UMR 5255, Université de Bordeaux, 351 cours de la Libération, Talence Cedex, 33405 France; 2 Laboratoire Interdisciplinaire Carnot de Bourgogne, UMR 6303 CNRS-Université de Bourgogne, 9 Avenue Alain Savary, Dijon, 21078 France

**Keywords:** Optical sensors, Surface chemistry, Optical spectroscopy

## Abstract

Bio-functionalized chalcogenide infrared optical glass fibers have been designed for evanescent wave mid-infrared spectroscopy. Surface biotinylation of the fiber tapered sensing zone has been achieved by reactivity of a maleimide function on sulfhydryl moieties of the glassy surface. Biotin-streptavidin interactions were studied by fiber evanescent wave spectroscopy. Kinetic measurement comparisons of functionalized and non-functionalized fiber surfaces for various protein concentrations have demonstrated the efficient bio-selectivity of the functionalized glass fibers. The protein enrichment of the functionalized glassy surface allows for a significant increase of the protein detection limit, greater than two orders of magnitude as compared to reference non-functionalized fibers. A detection of minute quantities at concentrations as low as 10 parts-per-billion is demonstrated. This study shows that bio-functionalized chalcogenide optical fibers allow to combine successfully surface bio-selectivity and infrared absorption fingerprints measurements to get a remarkable sensitivity enhancement of fiber evanescent wave detection methods in the mid infrared spectral range.

## Introduction

Optical biosensing and diagnostics are dominated by Surface Plasmon Resonance (SPR) and fluorescence-based approaches, which are very sensitive technologies but generally require sample labelling and provide no direct information on molecular structures. Molecular spectroscopy is an alternative approach with potential to provide a wealth of information on analyte composition, structure and conformation, allowing rapid and detailed biochemical analyses based on molecular fingerprints of biomarkers^1–5^. Chalcogenide glasses are inorganic vitreous materials that possess unique optical properties such as high non-linear refractive index, large transparency in the infrared (IR) optical range and their ability to be drawn into fibers^6,7^. The biocompatibility of these glasses is also demonstrated for the glassy compositions showing a long term stability in water^8^. All these features make these materials inescapable for designing IR optical systems aiming to record quantitative profiling of the fundamental vibration signatures of organic molecules in the mid-IR spectral region^9–11^. In this regard, the inherent molecular selectivity of mid-IR sensors characterizing the spectral fingerprint of chemical or biological species provides a good alternative as it is direct, fast, label-free and non-destructive^9,12^. Over the last decade, chalcogenide glass fibers have been successfully implemented in fiber evanescent wave spectroscopy (FEWS) experiments as it provides a real-time sensing without the cumbersome sample collection or pretreatment for the detection of biochemical species in various fields of applications including water pollution, microbiology, medicine and food safety^13–17^. Further, it provides a high degree of flexibility besides the advantage of the size of the probe which facilitates its insertion into chemical cells to follow reaction mechanisms^18^ as well as putting a section of an IR fiber optic probe in contact with living tissues to detect cellular metabolic activity, specifically cancer at early stages^19^.

A detection by FEWS technique is based on the evanescent field attenuation caused by the absorption of the surrounding media. The core of enhancing the sensitivity of a fiber sensor based on FEWS is to enhance the interaction between the optical evanescent field and the analyte^8,20,21^. One way is to enhance the intensity of the fiber evanescent field by optimizing the geometry/size of the optical guide; the second way is to increase the analyte concentration on the surface of the fiber evanescent field which can be obtained by specific surface functionalization of the optical material.

Regarding the first approach, a number of studies have been conducted in recent years on IR sensing based on tapered chalcogenide glasses. For example, Hocdé et al. have tapered Te-As-Se fibers from a diameter of 400 μm down to 100 μm to measure the concentration of acetone diluted in methylene chloride, they obtained values as low as 2.5%^7^. Le Coq et al. have employed Te-As-Se fibers tapered to a diameter of 50 μm to detect ethanol in water and a detection limit of 0.5% was acquired^22^. Velmuzhov et al. manufactured IR-tapered fiber sensors based on the Ge_20_Se_80_ composition with different geometries of the sensitive zone (straight, U-shaped form, one loop, two loops) to detect the content of an additive to diesel fuel and hence attained a detection limit of 0.02%^23^. Moreover, Yang et al. reported on tapering Ge-Se-Te glass fibers with a diameter of 400 μm to different taper waist diameters between 30 and 90 μm as sensors to detect mixed liquids containing different ratios of methanol and dichloromethane by FEWS^24^.

Compared to other sensing systems, mid-IR sensors are still limited in sensitivity to the high parts-per-million concentration levels due to the interfering absorptions of water, which presents a limiting factor especially for biological applications^25^. Detection at low parts-per-million and -billion concentration ranges necessitates functionalized surfaces allowing chemical or biochemical selectivity. This simultaneously enriches the targeted species within the volume probed by the evanescent field and minimizes the solvent absorption. For biosensors, receptors such as antibodies or aptamers can selectively increase the quantity of biomolecules targeted on the probe’s surface thus resulting in a drastic enhancement of the limit of detection.

The development of surface bio-functionalization methodologies for optical sensing is a classical strategy commonly developed on oxide glasses which are transparent in the visible/NIR domain^4,5^, however on IR optical material such approach is relatively limited in the domain of biosensing. Surface functionalization via short peptides, organosilanes or thiol monolayers have been demonstrated on Silicon^26,27^, ZnSe^28^, Germanium^29^, chalcogenide films^30^ and melt-quenched glasses^31^. Another method is to form a lipid bilayer containing nitrilotriacetic-acid on a Ge ATR crystal surface to characterize proteins by FTIR spectroscopy^32^. In the current paper, in order to demonstrate and quantify the advantage of a surface bio-functionalization for FEWS protein detection, we have pursued the work of Alvarado et al.^31^ on bio-functionalized sulfide chalcogenide glasses. Alvarado et al.. have studied three different approaches for the covalent grafting of biotin functional groups on a germanate sulfide chalcogenide glass : first, a traditional silane chemistry to functionalize hydroxyl moieties of the glassy surfaces, while other strategies were based on the reactivity of maleimide and cyclooctyne derivatives to react on sulfhydryl moieties. In the present work, the maleimide reactivity on surface sulfhydryl has been chosen for both its efficiency as well as for its simple one step procedure very well adapted to chalcogenide glass samples in the shape of mechanically fragile optical fiber.

Chalcogenide glass fibers based on a composition in the system Ge-Sb-S have been developed, tapered, and bio-functionalized using surface reactivity of thiol on the glassy surface. Finally, these IR fiber-optic probes were coupled to a FTIR spectrometer and tested for the bio-detection of protein solutions for various concentrations. A systematic comparison between non-functionalized and functionalized fibers has permitted to demonstrate the bio-selectivity of the probes and a quantification of the limit of detection improvement.

## Materials and methods

### Chalcogenide glass synthesis and fiber drawing

Chalcogenide glass synthesis of nominal composition Ge_15_Sb_20_S_65_ was prepared following the method indicated in Ref^31^. The preform was subsequently annealed for 4 h at 10 °C below its glass transition temperature (T_g_ = 250 °C).

The fabricated preform was then placed in the furnace and heated up to its softening point (460 °C) at a rate of 10 °C/min under helium gas flow (3 L/min) to be drawn into fibers using a dedicated 2 m high drawing tower. During the fiber drawing, the preform was slowly fed (2 mm/min) into the furnace as the drawing parameters were continuously adjusted in order to manufacture fibers with a controlled diameter ranging from 700 to 875 μm ± 5 μm, the average drawing speed was 0.3 m/min.

### Fabrication of fiber tapers

Short tapers were fabricated from fibers using a well-known post-processing technique based on VYTRAN Glass Processing Workstation (GPX-3400 series) device. The system consists of a filament heater, precision stages, with multi-axis control, and a microscopic high resolution CCD imaging system. As during the drawing process, the fibers were fed in the filament heater at constant speed, while being pulled at a higher speed on the other side. The high precision (1/4 µm) of the moving stages allows a fine tuning of the feeding and pulling speeds, and thus a fine control over the transitions shapes.

The taper manufactured for this work consists of two smooth transitions of 17.5 mm length reducing the fiber diameter on its middle and forming a uniform waist of 100 μm of diameter over a length of 60 mm.

### Biotinylation of the tapered fibers

Before each functionalization approach, all substrates were cleaned by sonication in CHCl_3_ for 15 min and dried under a N_2_ flow. Tapered fibers were immersed in a solution of 1.76 mL deionized water mixed with 405 µL of dimethyl sulfoxide (DMSO from Sigma Aldrich) and 34.4 µL of maleimide-EG3-Biotin (6.0 10^−3^ M in DMSO). The final concentration of Mal-EG3-Biotin is 9.4 10^−5^ M. DMSO is added to the solution to maintain the solubility of maleimide-EG3-Biotin in water. After 16 h at room temperature, the fibers were cleaned by immersion cycles in deionized water (2×) and CHCl_3_ (2×) using an orbital shaker.

### Bio-functionalization and in-situ bio-detection of Streptavidin via FEWS optical sensor

**Fig. 1:**
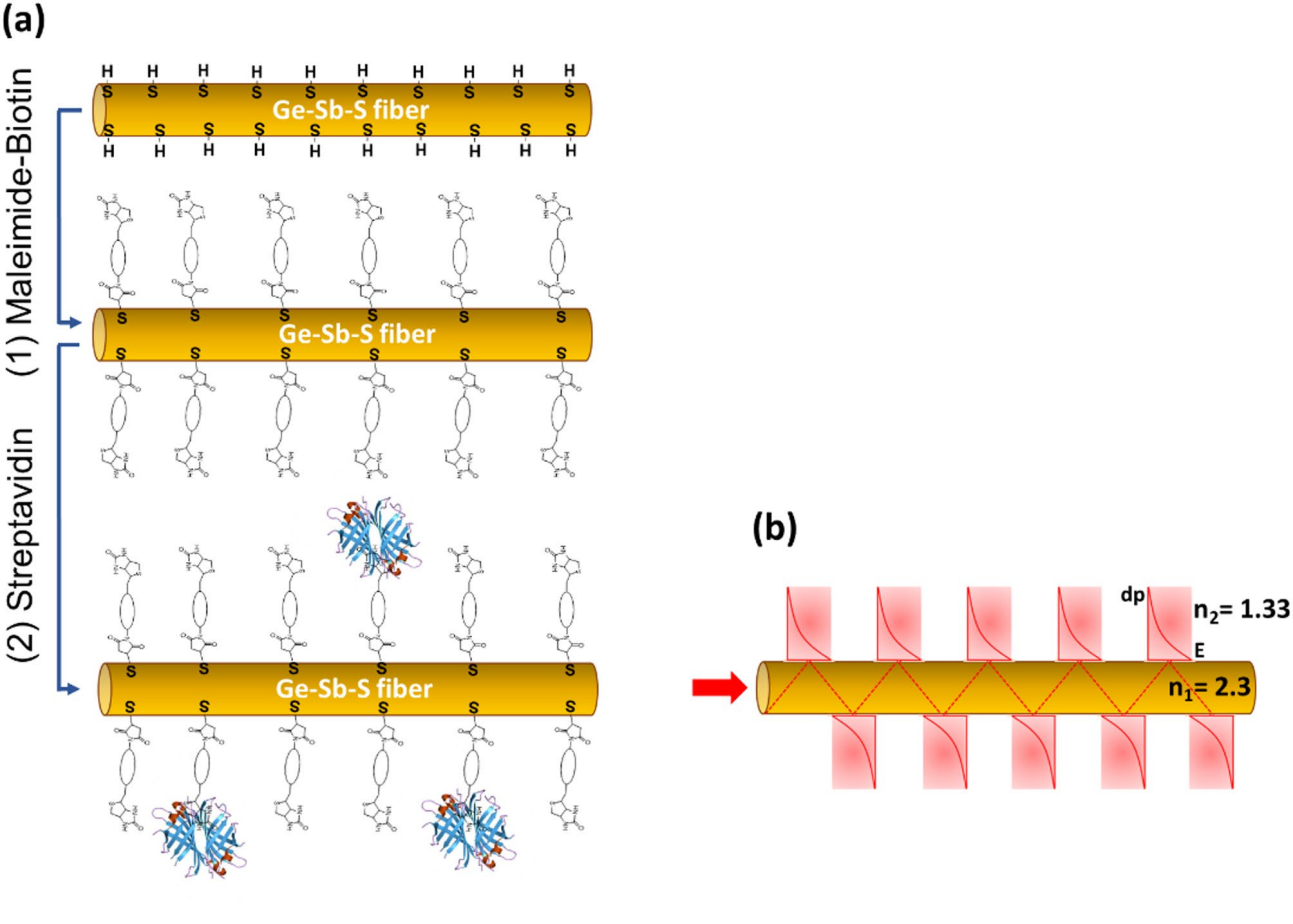
(**a**) Schematic representation of the functionalization approach on the fiber surface: (1) maleimide-based functionalization of sulfhydryl moieties to obtain a biotinylated fiber surface, and (2) surface bio-functionalization with Streptavidin in the presence of Tween 20 from Sigma/Aldrich Chemical Company. (**b**) Schematic representation of the FEWS approach.

**Fig. 2:**
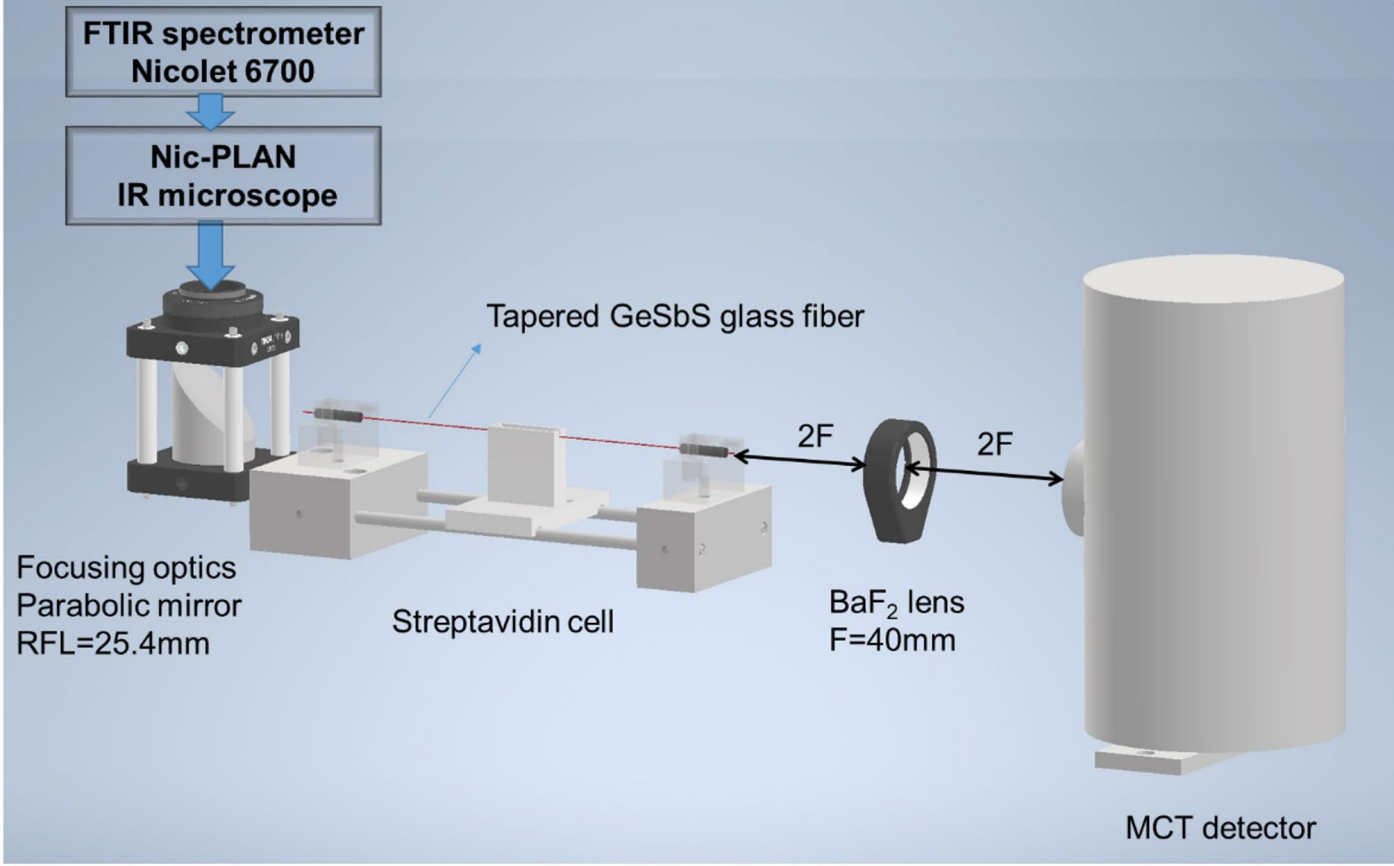
Experimental setup for the detection of streptavidin. This Figure was prepared using Autodesk Inventor 2024 (https://www.autodesk.com/).

The complete procedure followed to study biotin-streptavidin interaction at the surface of bio-functionalized chalcogenide fibers is illustrated in Fig. 1. The experimental setup used for FEWS experiments is presented in Fig. 2. It consisted of a microscope (Nic-Plan) attached to a FTIR spectrometer (Nicolet 6700) coupled to a parabolic mirror to inject the IR beam in the glass fiber which is maintain in position using classical fiber holders. All measurements were conducted for a room temperature of 22 °C (controlled using an air conditioner). The tapered fiber acting as a sensing probe is mounted on a 2 cm length polytetrafluoroethylene (PTFE) cell. The output signal from the fiber was collected using a biconvex lens BaF_2_ (F = 40 mm) in a 4 F optical system to focus the IR beam on a liquid nitrogen-cooled MCT detector. Initially, the cell was filled with PBS solution (Phosphate Buffer Saline, pH = 7.4) and a spectrum was recorded (t_0_). In a second step, the PBS solution was removed and the cell was filled with a solution of PBS-T (Phosphate Buffer Saline with Tween 20 from Sigma/Aldrich Chemical Company at 0.05% v/v, pH = 7.4) containing Streptavidin for three different concentrations 0.1 mg/mL (100 ppm), 10^−3^ mg/mL (1 ppm) or 10^−5^ mg/mL, (10 ppb). Tween 20 was used as blocking agent to limit non specific adhesion. To study the kinetics of the biotin-streptavidin interaction, seven to nine consecutive measurements were recorded every 15 min (named *t*_*i*_, where *i* = 1 to 9, for a total duration of 135 min). Each spectrum was obtained by averaging 2000 scans with a spectral resolution of 8 cm^−1^. The absorption spectrum of the analyzed sample was calculated using the following equation:

$$A(\bar{\upsilon})=-\text{l}\text{o}\text{g}\left(\frac{{I}_{S}}{{I}_{ref}}\right)$$

where $A (\bar{\upsilon})$ is the absorption at the wavenumber ῡ, $\:{I}_{S}$ and $\:{I}_{ref}$ are the intensities of light transmitted through the fiber located in an empty cell in the ambient atmosphere and immersed in the analyzed solution, respectively. All spectra have been normalized to the H_2_O stretching band at 3270 cm^−1^. Then, to characterize quantitatively the effect of streptavidin adsorption on the tapered fiber probe, the reference spectrum of the PBS solution has been subtracted giving the absorbance difference ($\:\varDelta\:A$).

## Results and discussion

As a reminder, Alvarado et al. have studied various reactivity approaches for the surface biotinylation of a sulfide glassy composition depending on the chemical functional groups observed on the glass surface: sulfhydryl (S-H) and/or hydroxyl (OH)^31^. In this study, the sulfhydryl reactivity with a maleimide chemical function has been chosen to functionalize the surface of chalcogenide fibers. Thus, using a commercial solution of Biotin-dPEG_3_^®^-Maleimide allows to obtain, by a simple and reproducible method, biotinylated sulfide glassy surfaces having a high affinity to streptavidin (STV) proteins. The impact on the detection of STV by FEWS IR spectroscopy of this particular method of surface bio-functionalization is presented in the following results.

**Fig. 3:**
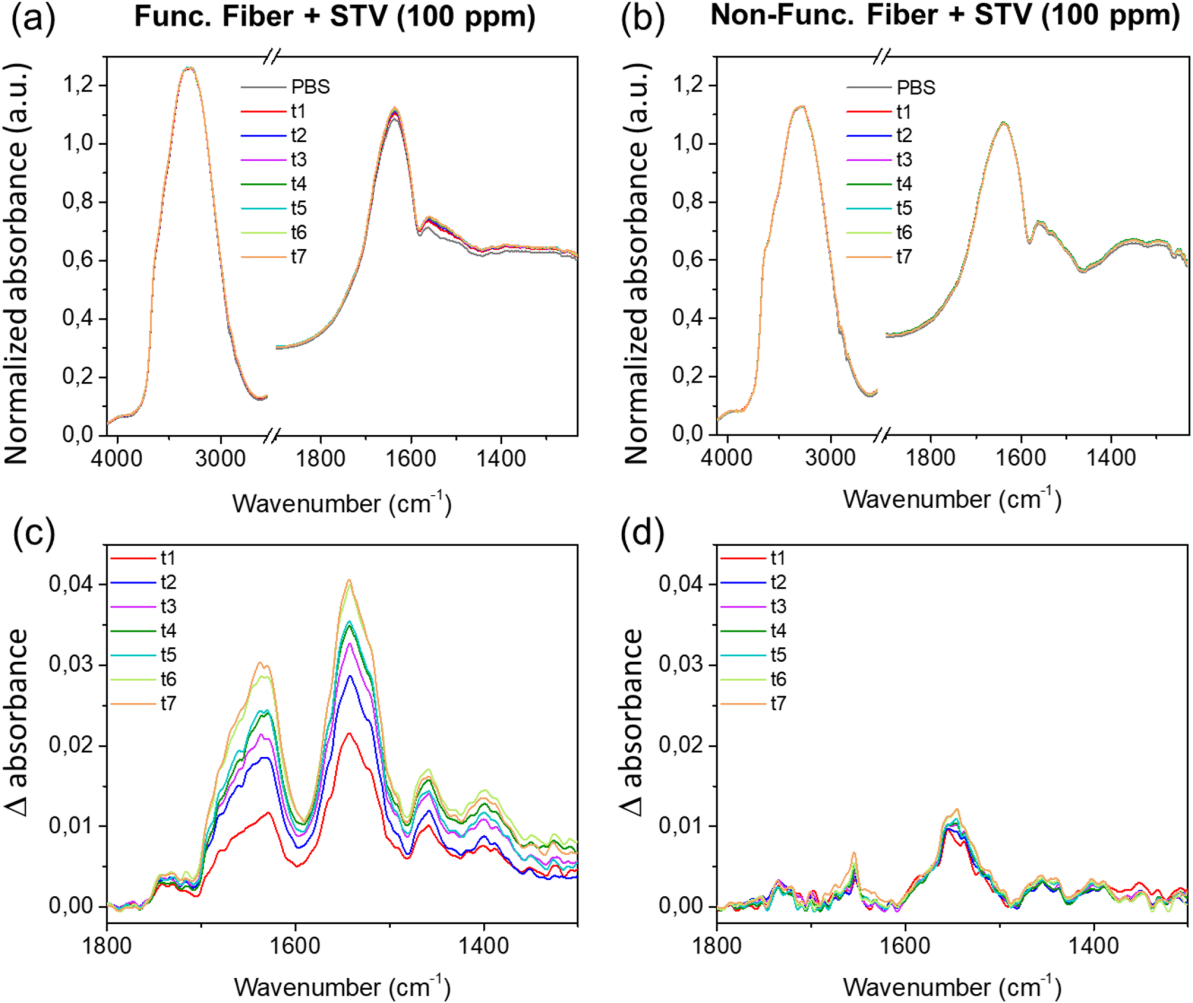
Evolution as a function of time of PBS-T containing streptavidin (high concentration of 100 ppm) absorbance measured by FEWS with (**a**) a biotinylated fiber surface and (**b**) a non-biotinylated surface. (**c**) and (**d**) show absorbance difference spectra obtained by the subtraction of the scaled buffer solution spectrum.

Figure 3 presents the spectra recorded after PBS-T containing-STV incubation (highest concentration of 100 ppm) as a function of time for the biotinylated and non-biotinylated tapered fiber surfaces. For all the spectra, the 4000–1500 cm^−1^ region is dominated by absorptions of water: OH stretching at 3270 cm^−1^, O-H bending at 1640 cm^−1^ and bend-libration combination band at 2100 cm^−1^. For a better visualization of the protein bands, we show insets in the 1690–1490 cm^−1^ range where amide I at 1633 cm^−1^ and amide II bands at 1540 cm^−1^ characteristic features of streptavidin can be detected. To better reveal the absorption bands of the protein, we have subtracted the scaled PBS spectrum from those of PBS-T containing-STV. Figure 3 (c, d) shows IR difference absorption spectra for biotinylated and non-biotinylated surface fibers. The presence of amide I, amide II, CH_2_ bending and amide III characteristic bands respectively at 1633, 1540, 1461 and 1407 cm^−1^ confirm the presence of proteins on the biotinylated surfaces of Ge_15_Sb_20_S_65_ glass fibers. One can see in Fig. 3 that the intensity of amide I, II, III and CH_2_ bending bands increase with the incubation time for the bio functionalized fiber probe whereas for the non-functionalized fiber the presence of STV is observed but do not change significantly with the incubation time. One can notice that in our optical configuration, the amide II band is more intense than amide I which is not the case if one compares to STV spectra measured by classical ATR configurations using high refractive index crystal of Si or Ge for example^26^. This can be attributed to strong band distortions caused by a perturbed total internal reflection configuration^33^. This is expected because of the relatively low refractive index of the GeSbS glass fiber (*n* ≈ 2.3)^34^.

**Fig. 4:**
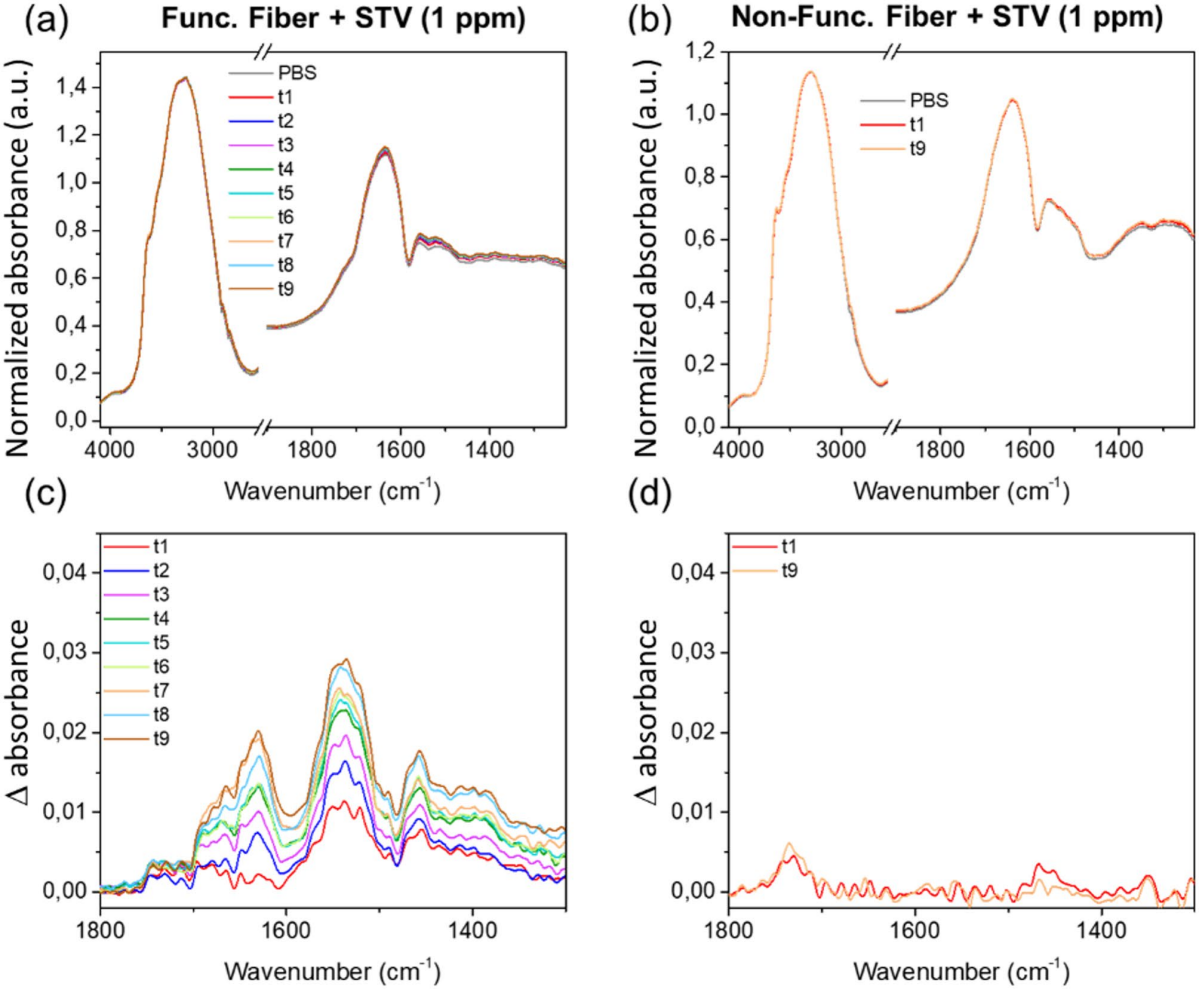
Evolution as a function of time of PBS-T containing streptavidin (1 ppm) absorbance measured by FEWS with (**a**) a biotinylated fiber surface and (**b**) a non-biotinylated surface. (**c**) and (**d**) show absorbance difference spectra obtained by the subtraction of the scaled buffer solution spectrum.

In Fig. 4, one can analyze the results obtained for the same method but using a PBS-T containing-STV solution at a concentration of 1 ppm for the incubation. For this lower concentration, the IR absorption bands attributed to the STV protein are clearly observed and increase with incubation time for the biotinylated fiber. In the case of the non-functionalized fiber, all the measurements present a band around 1732 cm^−1^ corresponding to carbonyl ester group attributed to Tween 20 which was used as a passivating agent to limit the non-specific streptavidin adhesion. One can notice that the Tween 20 IR absorption is detected for all the tested fibers. It shows an absorbance increase from approximately 0.0028 at t1 and remain stable after t2 with values around 0.0035.

These first observations prove the effective binding of the protein to the biotin groups on the surface of the functionalized fiber surfaces. This validates (1) the proof of concept for optical fiber surface bio-functionalization which allows the selective grafting of proteins and (2) the potential of lowering the detection limits to very low concentrations without the need to use concentrated protein solutions.

**Fig. 5:**
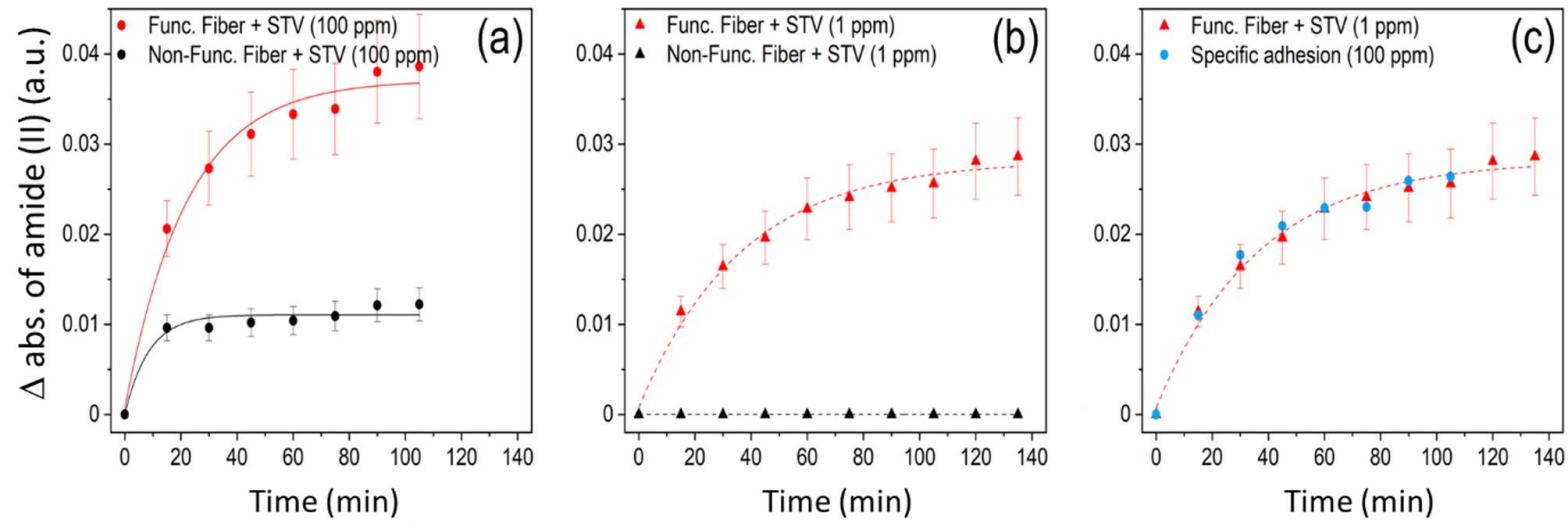
Binding kinetics of streptavidin to functionalized and non-functionalized surfaces: Plot of amide II absorbance as a function of time for biotinylated (red symbols) and non-biotinylated surfaces (black symbols) with (**a**) high (100 ppm) and (**b**) low concentrations of streptavidin (1 ppm). (**c**) Plot of the amide II absorbance corresponding to specific adhesion (blue solid circles), representing the subtraction of biotinylated from non-biotinylated surfaces in contact with streptavidin. On the same plot, we show again amide II absorbance for biotinylated surface in contact with low concentrations of streptavidin which represents only the specific adhesion (non-specific adhesion is not detected for 1 ppm concentration). Error has been estimated of 15%. Curves are only used as guide for the eyes.

In a second step, we will quantify more precisely the absorbance values linked to STV in the three cases. The amide II band was chosen since it is the most intense one. For the 100 pm concentration of STV in contact with the biotinylated surfaces (Fig. 3c), absorbance values increase from 0.02 at t1 to 0.04 at t7 (Δ = 0.02). Employing the same concentration but with non-biotinylated surfaces (Fig. 3d), the absorbance of amide II ranges from 0.0096 at t1 to 0.012 at t9, the STV signal is clearly observed but its time variation is within the error of the measurement, and thus can be considered as almost constant from t1 to t9 for this experiment. The STV detection observed for the non-biotinylated fiber corresponds to the non-specific adsorption which is detected only for the incubation with the highest STV concentration. For the 1 ppm concentration of STV in contact with biotinylated surfaces (Fig. 4c), absorbance increases from 0.011 at t1 to 0.029 at t9 (Δ = 0.018). For more details, the kinetics of STV detection is plot in Fig. 5 for the four cases described above. In Fig. 5a, we present the evolution of the amide II absorption for a biotinylated fiber and a reference non-functionalized fiber exposed to the same 100 ppm protein concentration. The kinetics for the former case is much slower than the latter one. This suggests that, in the absence of chemical functionalization by biotin, the non-specific adhesion of STV on the fiber surface occurs within the first 15 min and then do not evolve significantly. In contrast, in the presence of a chemical surface functionalization, the protein detection stabilizes after 100 min. Figure 5b depicts the amide II absorption for 1 ppm STV concentration for both functionalized and non-functionalized fibers. No protein has been detected on the non-functionalized fiber (Fig. 4d), indicating that the non-specific adsorption occurring at this concentration is under our limit of detection. However, for the biotinylated fiber, we evidence a clear gradual increase of the amide II adsorption. Finally, in order to compare the specific protein adsorption occurring on biotinylated fibers for 100 ppm and 1 ppm STV concentrations, we have estimated the part of specific adhesion for the kinetic measurements done for the 100 ppm STV concentration by subtracting the absorbance of STV measured for the reference non-functionalized fiber (showing only non-specific adsorption) from that of the biotinylated fiber (showing both specific and non-specific surface interactions). The estimated kinetics of specific detection of STV for both concentrations are plotted in Fig. 5c. One can notice a very good correlation of the data demonstrating that for a concentration above 1 ppm of STV, the strong affinity of the fiber surface allows to obtain a similar saturation coverage of the surface independently of the protein concentration. This is explained by a STV concentration at the probe surface governed by the surface bio-functionality and not limited by the STV volumetric concentration and diffusion processes. This confirms the validity of the present chemically functionalized optical system which permits an effective immobilization of proteins at the surface of the fiber probe.

**Fig. 6:**
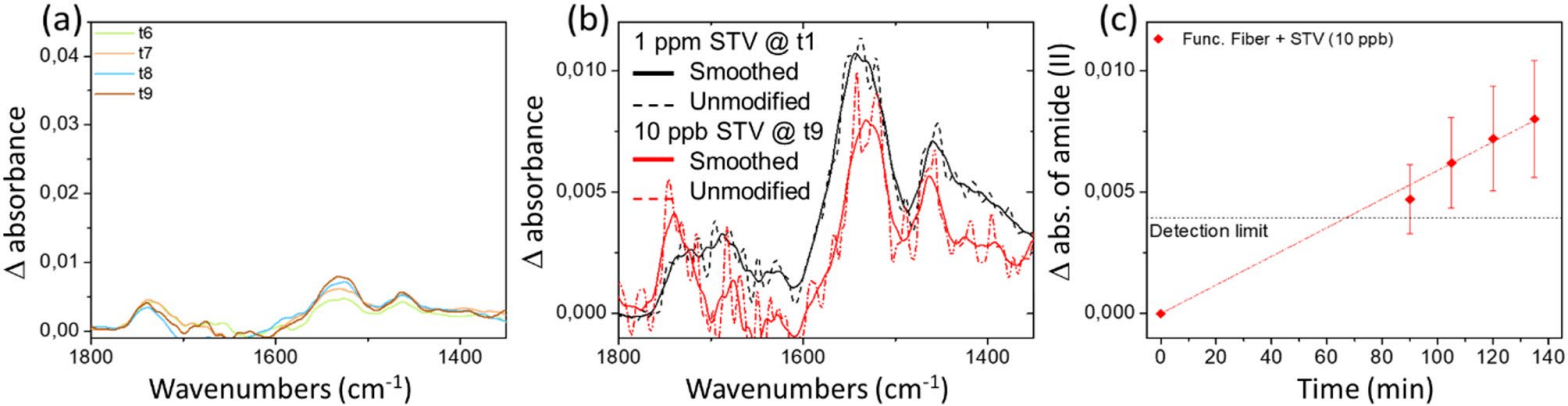
(**a**) Evolution as a function of time of absorbance difference spectra obtained by the subtraction of the scaled buffer solution spectrum to the PBS-T containing streptavidin (10 ppb) absorbance measured by FEWS with a biotinylated fiber surface. (**b**) Comparison of absorbance difference spectra measured for 1 ppm concentration after 15 min of incubation and the one measured for a 10 ppb concentration after an incubation of 135 min. Unmodified and smoothed data are respectively presented in dashed and full lines. (**c**) Absorbance kinetics of the amide II band during 135 min of incubation in a 10 ppb PBS-T containing STV solution.

After the successful demonstrations of protein detection and bio-affinity of the functionalized chalcogenide fibers developed, we have decided to test a much lower STV concentration of 10 parts-per-billion. For this purpose, we have diluted the concentration already analyzed (0.001 mg/mL) by a factor of 100. As shown in Fig. 6, the spectrum of streptavidin has been successfully detected but only after an incubation of 80 min. The detection limit was determined as follow: the maximum of the absorption band of amide II (@1540 cm^−1^) should be 2 times greater than the noise level of the measurement. In the specific case of the 10 ppb STP sensing, the noise level observed on the unmodified spectra is a absorbance difference of Δabs = 2.10^−3^. Thus the noise level was set as Δabs = 4.10^−3^. One should notice that this detection limit can vary from one experiment to another as the amount of IR light injected in the fiber and collected by the detection unit can vary. For longer durations, the absorbance of STV increases linearly and reach for t9 an absorbance close to 10^−2^ equivalent to the one observed at the early time of the kinetics done for the 1 ppm STV concentration. It is expected that the rate of protein adsorption on the biotinylated fiber surface for a such low concentration is not only governed by the surface properties, but is diffusion limited because of the weak presence of proteins in the vicinity of the fiber surface^35^. This last set of data demonstrates the strong advantage of using biotinylated chalcogenide glass fiber surface for the use of IR FEWS spectroscopy as a bio-detection method. It has been shown that a pristine fiber does not allow the detection of STV at 1 ppm concentration, whereas STV detection is achieved at 10 ppb for a functionalized chalcogenide fiber probe. This represents an improvement of at least two orders of magnitude. Nevertheless, one should notice that such long incubations induce some risks of degradation for real bio applications, it points out that some efforts will be needed to accelerate diffusion limited surface adsorption.

As compared to the literature results of FEWS Mid-IR sensor, the surface functionalization used in our study has permitted a considerable improvement of the method sensibility. As example, the lowest detection limit, to the best of our knowledge, was reported by Velmuzhov et al.^23^. They have manufactured chalcogenide IR-tapered fiber sensors with different geometries of the sensitive zone to attain a detection limit of 0.02% (# 200 ppm) of an additive to diesel fuel^23^. It confirms that the analyte enrichment at the surface of the chalcogenide fiber sensor using selective chemical functionalization is very effective for FEWS and can improve the detection limit of such sensing approach by more than 2 orders of magnitude. At this point, one should also compare our results to other IR spectroscopic methods not using an optical fiber as sensing probe^36,37^. A first good example is the findings of Hinkov et al.^38^ on monitoring biomolecular interactions in a dynamic fashion using an integrated microfluidic device. They achieved very low detection limits for different biomolecules, as low as 0.075 mg/mL. Their approach highlights the particularity of the device’s design to improve signal-to-noise ratio. Furthermore, Kratz et al.^39^ have employed an optofluidic system as well as surface chemistry functionalization to enhance IR absorption using surface enhanced IR absorption (SEIRA) on a metal island film, attaining detection limits of streptavidin at the nanogram scale. Similarly, Campu et al.^40^ demonstrated the capability of gold nanpyramids to enhance significantly the IR absorption signal to develop ultrasensitive SEIRA-based immunosensors, exhibiting a detection limit of streptavidin of 1 pM. Vrážel et al.^41^ have introduced a real-time monitoring to detect organic pollutants in water using a chalcogenide IR photonic sensor functionalized with a polymer membrane. The sensor demonstrated sensitivity enhancement with a detection limit of 100 ppb. Compared to previous studies, our work demonstrates comparable, if not superior sensitivity, using tapered biofunctionalized fibers, achieving detection limits down to 0.189 nM (10^−5^ mg/mL). This suggests that our setup provides strong surface interactions for enhancing sensitivity. One should mention that direct comparison is challenging due to differences of experimental setups and in some cases of target bioanalytes. Nevertheless, the ability to detect such low concentrations demonstrates the efficacy of our tapered functionalized fiber approach.

Finally, our results are very encouraging but there is still some efforts to compete in terms of limit of detection with electrochemical, fluorescent or SPR based optical sensing for which a ppb level of detection or lower is currently achieved^1–3^. To further improve the sensitivity of FEWS Mid-IR sensor, one could increase the sensing optical length or optimize the evanescent wave using different fiber geometry. Nevertheless, if one aims sensing in aqueous media, we are always limited by the strong water absorption (absorbance above 1 for a 2 cm sensing length in our case). Thus, one should not expect a large improvement by optimizing the sensing fiber geometry. However, the signal-noise ratio can be considerably improved by increasing the amount of light injected in the fiber in order to increase the signal level on the detection. This could be achieved by changing the light injection method or by using a new kind of IR source with a higher brightness, such as quantum cascade technology. In addition to that, one can consider agitation or microfluidic systems to mitigate diffusion limitations. This proof of concept clearly opens new opportunities for using chalcogenide glasses combined with surface functionalization methods in biological analysis and detection by infrared spectroscopy.

## Conclusions

Chalcogenide IR fibers are suitable for evanescent wave spectroscopy especially because of the possibility to obtain increased sensitivity in tapering the fiber. We experimentally demonstrated using FEWS that the proposed sensing bio-functionalized architecture confirm the adsorption of bioanalyte by providing on one hand a chemical identity of the targeted molecules from their IR absorption fingerprints, and on the other hand providing a remarkable enhancement of the sensitivity of detection. Such fingerprinting sensitivity enhancement may help to identify bioanalytes in biochemical sensing, especially when the analyte quantity is very limited. Hence, this novel bio-functionalized chalcogenide tapered fiber can be highly promising for future diagnostic point-of-care assays.

## Data Availability

The authors declare that the data supporting the findings of this study are available within the paper. Should any raw data files be needed in another format they are available from the corresponding author upon reasonable request. Source data are provided with this paper.
